# Improving our model of cascade testing for hereditary cancer risk by leveraging patient peer support: a concept report

**DOI:** 10.1186/s13053-021-00198-7

**Published:** 2021-09-26

**Authors:** Suzanne C. O’Neill, Jada G. Hamilton, Claire C. Conley, Beth N. Peshkin, Rosalba Sacca, Glynnis A. McDonnell, Claudine Isaacs, Mark E. Robson, Kenneth P. Tercyak

**Affiliations:** 1 Georgetown Lombardi Comprehensive Cancer Center, Washington, D.C, USA; 2 Memorial Sloan Kettering Cancer Center, New York, USA

**Keywords:** Cascade testing, Family communication, Intervention, Peer coaching

## Abstract

Consensus and evidence suggest that cascade testing is critical to achieve the promise of cancer genetic testing. However, barriers to cascade testing include effective family communication of genetic risk information and family members’ ability to cope with genetic risk. These barriers are further complicated by the developmental needs of unaffected family members during critical windows for family communication and adaptation. Peer support could address these barriers. We provide two illustrative examples of ongoing *BRCA1/2*-related clinical trials that apply a peer support model to improve family communication and functioning. Peer support can augment currently available genetic services to facilitate adjustment to and effective use of cancer genetic risk information. Importantly, this scalable approach can address the presence of cancer risk within families across multiple developmental stages. This applies a family-centered perspective that accommodates all potentially at-risk relatives. This peer support model can be further applied to emerging topics in clinical genetics to expand reach and impact.

## Background

### The promise of cascade testing

Cascade testing refers to the receipt of genetic counseling and testing among blood relatives of individuals (i.e., probands) with specific pathogenic/likely pathogenic germline genetic variants (i.e., mutations), allowing them to pursue appropriate cancer screening and risk reduction strategies. This approach can significantly expand the benefit of cancer genetic testing, particularly for high risk pathogenic gene variants [1]. Relatives who are younger and cancer unaffected would stand to gain the greatest health benefits. Several clinical guidelines recommend the use of cascade testing [2–5], emphasizing the importance of effective strategies for supporting family communication and the understanding of risk information within families [6–8].

**Fig. 1:**
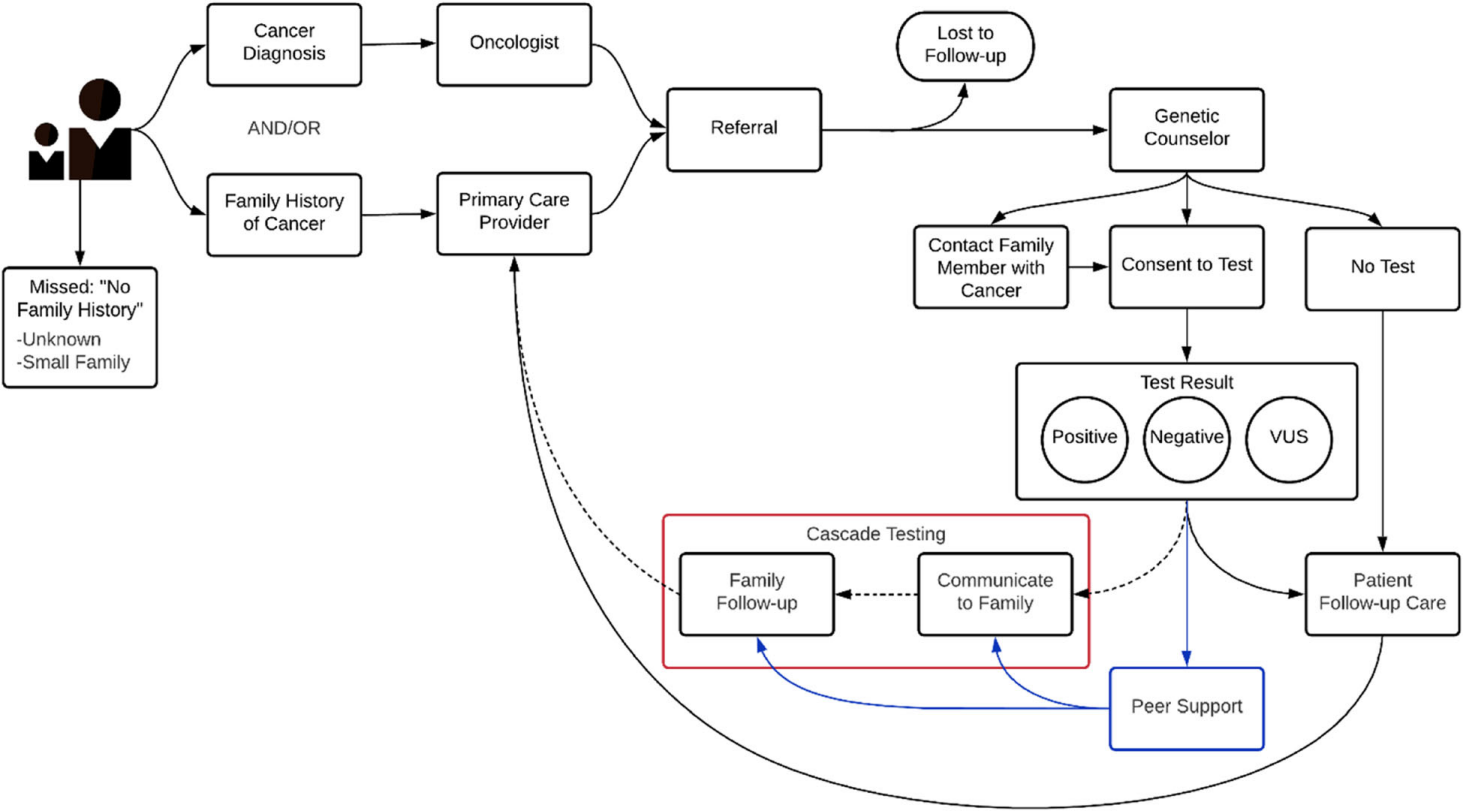
Process of genetic counseling and testing. Adapted from Helzlsouer (2018). The red box highlights the process of cascade testing, dashed lines indicate breakdowns in cascade testing. The proposed role of peer support is indicated in blue. VUS = variant of uncertain significance

Despite its promise, several barriers remain to effective cascade testing in routine care or clinical research. Figure 1 [9] depicts the process of genetic counseling and testing; breakdowns in the process of cascade testing are represented with dashed lines. First, the specific genetic result identified within the family must be successfully communicated to the family members for whom it could have medical implications. Standard of care includes methods to support the proband’s dissemination of positive test results, often in the form of a family letter detailing the specific mutation(s) in their family and related information. However, varying levels of familial contact and challenges associated with the timing of this information (e.g., delivering it in the context of a cancer diagnosis, relevance to younger family members who could maximally benefit from the information, cultural barriers in sharing information about cancer diagnoses), limits the efficacy of this standard approach. Second, awareness is often not consistently translated into action among family members [10]. Finally, in addition to genetic risk, communication patterns and ways of coping, both effective and ineffective, are also transmitted within families. Studies of family communication show varying rates of communication of genetic risk information, ranging from almost universal to minimal or no communication [11–15].

Connecting family members with cancer risk information without the skills to take the necessary next steps can potentially lessen the long-term impact of the information on personal, familial, and population health [16]. For example, an at-risk adult relative must be prepared to cope emotionally with the information and carry out the process of risk assessment and management. Further, to ensure full intergenerational benefits (e.g., cancer prevention), carriers with biological children must be able to communicate openly with their potentially at-risk adolescent and young adult offspring using language appropriate to their needs and circumstances [13]. Effective cascade methods must integrate these intergenerational and intrafamilial variables and connect them with promising methods for intervention that are scalable into routine clinical and community care [17].

Traditionally, these activities are initiated by members of the healthcare team. Yet, the resources offered within the healthcare system may not be sufficient to also meet the information and support needs of probands and their family members [18]. Additionally, not all healthcare providers have the time and/or expertise to adequately support patients in forming and carrying out the behaviors required of a testing cascade. One possible solution to these challenges is to provide patients with a structured interaction with a trained lay peer supporter [19, 20]. Peer supporters could provide role modeling and coping skills training to bridge gaps in effective cascade testing. Critically, this peer-to-peer support approach can also address the presence of cancer risk within the family across multiple developmental stages and embrace a truly family-centered perspective that accommodates all potentially at-risk relatives [21]. Peer supporters can provide training in effective methods of communication, decision support, and movement towards actions in support of addressing cancer risk [22]. This model can extend current standard of care by both supporting the proband and allowing family members to (re)engage with cancer risk information at critical windows for awareness and action without overburdening the genetics workforce.

## Peer support to augment clinical genetic services

We are currently investigating ways to fill the gaps between recognized needs and available resources using the scalable approach of peer support. Peer support leverages the shared lived experiences of individuals to allow a supporter to help similar others to acknowledge their healthcare needs, seek clinical care, and manage health conditions effectively [23]. Most peer support programs connect newly-diagnosed individuals with those who have been managing their disease for some time. In the context of disease management, peer support can be structured around definitive clinical guidelines that newly-diagnosed individuals can learn to adhere to in order to improve their health outcomes. Examples of this would be long-established programs for diabetes and hypertension [24] as well as the broader field of community health workers [23].

Applying peer support for those with hereditary risk of disease would vary from the traditional model in important ways. Support for genetic testing and risk management among cancer-unaffected patients can include guideline-informed care decisions, but must integrate the preference-sensitive nature of the decision to test and to then manage risk. In the case of genetic risk, peer support can provide a model of lived experience, but must balance this with the many options for effective decision making and risk management. Peer support would supplement both clinical care provided by genetic counselors and other specialists, as well as informal support from family who have a shared experience of disease risk.

In two ongoing clinical trials, we are collaborating with highly motivated, trained lay volunteers who have been identified as being at high risk for cancer themselves and want to assist others in navigating this journey. Below, we provide further detail regarding how peer support can be uniquely applied to facilitate adjustment to, and effective use of, cancer genetic risk information.

## Two examples of providing peer support for ***BRCA1/2*** carriers and their families through a developmental lens

*BRCA1/2* gene mutations confer ≥ 85 % lifetime breast cancer risk and a 13 %-46 % lifetime ovarian cancer risk [25]. Adult *BRCA1/2* mutation carriers have surveillance and risk-reduction options to manage their elevated risks [26]. Most women who undergo *BRCA1/2* genetic testing believe the information can be used in proactive medical decision making, both for themselves and their relatives [27, 28]. Family communication about a *BRCA1/2* mutation is recommended by numerous national organizations so that blood relatives can learn about and manage their own cancer risk through genetic counseling and testing [29–31]. The Centers for Disease Control and Prevention has highlighted *BRCA1/2* as a top priority for cascade screening because testing may lead to decreased morbidity and mortality if proactive steps are taken for early detection and risk reduction [32]. However, *BRCA1/2* genetic testing is not recommended in childhood due to the lack of immediate medical implications and concern over its psychosocial strain [31, 33]. Thus, decision making about testing is usually deferred until adulthood (usually in mid-20’s or later).

Despite the recommended delay in testing, there are several windows of opportunity to promote communication about familial cancer prevention with minor children [34]. For example, mothers who are *BRCA1/2* carriers are often keenly interested in knowing their children’s cancer risk, especially those presenting for testing out of concern for their children’s future health. In studies of mothers undergoing *BRCA1/2* testing, stronger tendencies to worry/ruminate about cancer risk information, greater psychological strain, and poorer coping skills are identified as barriers to open family communication [35–38]. Thus, *BRCA1/2*-positive mothers of adolescent and young adult children may benefit from behavioral intervention by their peers–addressing mothers’ unique concerns and questions about ways to communicate hereditary cancer risk information to their offspring.

Young adult children may also benefit from peer support interventions. Although they may pursue cascade testing, the rate of clinical uptake in this population is low. For males who test positive, there are no medical implications during this time frame. For females, if positive, guidelines recommend screening with clinical breast exams and breast MRI beginning at age 25. Some young women may consider altering their use of oral contraceptives or child-bearing, and some consider undergoing risk-reducing mastectomy before age 25. Further, all at-risk young adults may seek genetic testing for reassurance or to reduce uncertainty.

Our work with young women, aged 21–30, indicates that they have unique support needs as they enter the window when they would be considering genetic counseling, testing, and risk management. Patterns of testing over the past decade have shifted to include more younger and cancer-unaffected women [39]. However, counseling, testing and risk management of young women present several clinical dilemmas related to the timing and choices of risk management through screening or surgery. The potential impact of risk management on quality of life and family planning must be balanced against the low 5-year breast cancer risk estimates that most young women face [40–42]. Decisions about counseling, testing and risk management take place in the context of less decision-making experience [43] and the developmental tasks of young adulthood, such as career planning, selecting a mate, and family planning [44, 45]. Further, decision processes of young adults are more prone to affective biases than those of their older counterparts [46]. As such, decisions could be motivated by the need to reduce uncertainty and distress [47]. This relief could be temporary as the need to make health decisions emerges [48, 49]. Prior work by our group suggests approximately half of these young women experience clinically significant levels of cancer-related distress. This distress was not significantly associated with tested status, underscoring the range of challenges facing this young population. However, distress was associated with young women’s report of lower peer social support and information satisfaction, and greater familial disruption and perceived cancer risk [18], indicating subgroups of women in specific need of support.

In order to maximize the impact of cascade testing, the types of peer support that are offered should be tailored to acknowledge the familial nature of communication and integrate the developmental needs of those within the family. We currently are testing two strategies to use peer support to improve outcomes among at-risk families (Table 1), each targeted to the developmental needs outlined above.

### Parent Communication Study (PCS)

We assert that open communication among mothers who are *BRCA1/2* carriers and their families is essential to cascade testing. In an ongoing trial (ClinicalTrials.gov Identifier: NCT04258280), our goal is to improve cancer genetic counseling outcomes for *BRCA1/2*-positive mothers and their adolescent and young adult (AYA) children. We do this through a theoretically-grounded and evidence-based decision making/structured peer support program called “My Children, My Test Result & Me.” By incorporating peer support and decision skill-building into a behavioral intervention, we seek to facilitate maternal psychosocial adaptation and disclosure of *BRCA1/2* status -- precursors to cascade testing. Key outcomes in this trial are self-reported maternal disclosure to AYA children, social support enhancement, and decisional conflict and psychosocial distress reduction. Augmenting standard genetic counseling, our main hypothesis is that our intervention improves maternal communication, informed decision making, psychosocial distress, and interactions between mothers and their AYAs, with each of these outcomes assessed with valid measures. Mothers receive either “My Children, My Test Result & Me” (intervention condition) or our decision guide alone with standard care (control condition). Our decision guide provides information about talking to AYAs about hereditary breast cancer–educating parents about ways to discuss hereditary cancer risk with children. The decision guide also provides referrals to externally-managed peer support programs, with toll-free telephone numbers and no-cost navigation services.

Our trial will be among the first to directly monitor adaptation and disclosure processes and outcomes among mothers with *BRCA1/2* mutations and their AYA children. Our work drives the field forward with a fully-manualized, peer-to-peer, decision support intervention that would be poised for dissemination and implementation in clinical and community settings, and adds to the scant literature on the utility of cancer genetic peer support programs. Trial data are necessary to inform clinical issues raised in differentiating between communicating with adolescents about family history and genetics vs. testing adolescents for *BRCA1/2* (which is both controversial and contraindicated for the vast majority of adolescents). Our trial also breaks new ground on the ways in which intergenerational genetic counseling prepares mothers and AYAs for their genetic futures.

### Peers and Cancer Empowerment (PeACE)

 Young adulthood is a critical time to increase awareness of cancer risk, prepare for cancer genetic counseling and testing, and integrate results into healthcare decision making. Ensuring that young adults in hereditary cancer families have effective means to learn healthy coping strategies to manage the unique communication and decision-making challenges they face is essential to leveraging the value of cascade testing. Learning these skills from a peer could further fill the void often felt by young people who lack peers who share their specific health concerns [20, 50, 51]. Indeed, several community organizations that support the hereditary cancer community have worked to fill this gap and offer some type of peer support program. Combining health information and peer support with a brief coping skills training program using a theoretically-grounded, evidence-based approach that attends to the unique developmental needs of young adults could prove a powerful and disseminable approach.

We developed a new, fully manualized/scripted intervention called “*Peers and Cancer Empowerment* (*PeACE*)” to meet the unique psychological, developmental, and medical needs of young women in *BRCA1/2* families. This three-session, telephone-delivered, peer-led coping skills intervention is structured around three primary needs identified by our earlier work: communication skills oriented towards family and care providers, health decision making, and managing emotions. In our ongoing trial (ClinicalTrials.gov Identifier: NCT04248257), the PeACE intervention is compared to community-based peer support provided by several community organizations that serve this population. Outcomes include distress, decisional conflict, and uptake of counseling and testing among untested young women. These are assessed via self-report measures using validated measures, with the use of counseling and testing confirmed via the clinical record. Participants complete assessments at baseline, as well as 1, 6 and 12 months after coaching. Use of risk management approaches will also be tracked; given the age of our participants, these are not trial outcomes.

### Shared structure of the PCS and PeACE trials

For each of these trials, peer coach interventionists are lay individuals (mothers or young women) who are compensated for their time. Parent coaches are mothers who are *BRCA1/2* carriers who are at risk for, or surviving with, hereditary breast cancer. Peer coaches are young *BRCA1/2* carriers, recruited from the community. All coaches participate in group training sessions and ongoing supervision within our research team. Group training includes: (1) review of *BRCA1/*2 genetic counseling and testing issues, (2) human subjects research and privacy protections, (3) socialization with other parent/peer coaches, (4) an overview of the intervention and coaching role, (5) coaching interaction style guidelines (e.g., confidentiality, rapport building, active listening, crisis/emergency situations), (6) role-playing peer mentorship skills, (7) an overview of participant assignment, intervention call structure, and expert supervision, and (8) additional IRB trainings as required.

Emphasized throughout training is their decision support and peer support role as the parent/peer coach, and the distinction between this role and that of a clinical provider. Coaches are trained to not give any medical advice and to refer participants requesting cancer risk management information (e.g., screening, surgery) to the participant’s clinical team. Participants are also informed of the limitations of the role of their assigned parent/peer coach. Parent/peer coaches complete an online survey detailing each interaction with their participants prior to supervision. Coaches are expected to possess strong interpersonal and telephone skills, a nondirective attitude, good active listening, verbal communication, and empathy (as demonstrated during training and evaluated by the study team). Coaches are trained to mastery on the scripted intervention: mastery is verified against an intervention performance checklist that includes critical parent/peer coach behaviors. After reaching mastery, the checklist is continuously applied to a random sample of recorded intervention sessions. We anticipate that the rigorous training approach used in these trials will not only ensure the faithful delivery of these interventions, but will also provide valuable lessons and tools that can ultimately be applied to future efforts to deliver peer support at scale.

**Table.1:** Content and process of the PCS and PeACE peer coaching protocols

	Goals/Content	
**Call**	**PCS**	**PeACE**
**1**	• Clarifying potential communication choices that mothers have (e.g., share all, some, or no information with their children). • Identifying how values may be derived from multiple sources, including maternal psychological stress reactions/responses to their genetic testing, concern for family communication and well-being, extent to which they value input from spouses/partners, friends. • Addressing how maternal values and preferences may manifest in decision making needs, including family-of-origin beliefs, cultures, systems.	• Introduction and orientation to the program and working with a peer supporter. • Recognizing emotional, cognitive, and behavioral sources of hereditary cancer stress. • Presentation and practicing of problem solving approaches and the content of improving communication, making decisions, and managing distress. • Navigating the use of the participant workbook to apply problem-solving. • Assign homework that applies problem-solving techniques.
**2**	• Exploring issues and needs in communicating genetic test results to children and managing stress: o Patient types (previvor, newly diagnosed, survivor) o Implications of risk o How to assess children’s readiness to learn about hereditary risk o Communicating risk to children based on child age/gender o Pros/cons of communicating risk o Considerations for parents who choose not to communicate with children about BRCA • Using interactive decision guide worksheets to explore decision making.	• Review of homework problem-solving techniques. • Reinforce coping strategies: o Focusing on ways of enhancing coping and decreasing distress o Identifying healthy and unhealthy coping strategies o Assess information needs o Apply cognitive problem-solving training to hereditary cancer risk • Assign homework that applies problem-solving techniques.
**3**	• Discussing risk with children if/when the time is right, emphasizing if/how plans meet preferences/values. • Reviewing communication strategies/conversations that may occur–including consideration of vocabulary and terminology, explaining inheritance, risks, and management options, genetic testing in adulthood, psychological concerns. • Assessing children’s reactions, helping them cope with/assimilate hereditary risk. • Referencing useful websites and books. • Reviewing completed worksheets.	• Enhancing risk comprehension, decision making and managing emotions. • Specific steps to take to gain information and support about hereditary cancer risk, working with how to anticipate their thoughts and feelings, making plans for next steps. • Use of vignettes/role-playing techniques, problem-solving, communication, decision making skills training, managing emotions, reinforce resource utilization.

## Additional opportunities to leverage peer support to improve cascade testing outcomes

While the strategies discussed above apply peer support to genetic counseling and testing for *BRCA1/2* mutations, these approaches could be translated to other genomic applications. First, multigene panel testing is currently recommended over limited *BRCA1/2* analysis for patients with suspected hereditary breast and ovarian cancer (HBOC) syndrome [52–55]. Multigene panel testing efficiently assesses for variants in both *BRCA1/2* genes and other rarer moderate- or high-penetrance “non-*BRCA*” genes (e.g., *PALB2*, *CHEK2, ATM*) [56]. Second, HBOC is defined by the Centers for Disease Control and Prevention as a Tier 1 Genomic Application. This designation indicates that early detection and intervention has significant potential for positive impact on public health based on available evidence-based guidelines and recommendations [5]. Other Tier 1 Genomic Applications, familial hypercholesterolemia and Lynch Syndrome, would be a logical extension of peer support models for genetic counseling and testing. The developmental lens described above may be particularly appropriate to apply to both of these. In the context of Lynch Syndrome, screening for carriers may begin as young as age 20 [4], while several clinical guidelines recommend universal screening for familial hypercholesterolemia for all children [57] as well as lipid management [58]. A third application that includes these examples and others include the 59 genes noted as actionable by the American College of Medical Genetics and Genomics (ACMG) [59]. Peer support models might be leveraged to support decision-making about testing for these high-risk genes.

In addition, the peer coaching model described above could be delivered in other contexts and formats to address workforce issues [60] and support widespread dissemination. Given the swift pace of development in communication technologies, there has been increasing interest in mobile health (mHealth) intervention approaches to support cancer prevention and control. mHealth technologies include mobile phones, smartphones, text messaging, social media, patient monitoring devices, and other wireless devices. Some peer coaching interventions have incorporated mHealth technologies including wearable sensors, smartphone applications, and social media [61, 62]. Although the evidence for the efficacy of mHealth interventions is limited [63], combining peer support and mHealth technologies has the potential to produce effective and scalable interventions in cancer prevention and control. Integration of mHealth approaches may be particularly powerful for younger cohorts; 98 % of U.S. residents ages 18–36 report using the internet at least occasionally or owning a smartphone [64]. Likewise, given the recognition that there is often a span of time between initial awareness of the presence of genetic risk and readiness for action, peer supporters could serve as both a more feasible and potentially more acceptable way to keep at-risk individuals engaged with the need for future clinical care when these individuals initially decline testing.

The motivation of those within the hereditary cancer community to volunteer as peer supporters is suggested by the presence of several peer support programs within notable community organizations that serve the hereditary cancer community. The widespread use of peer supporters within these organizations speaks to the potential scalability of offering the skills-based coaching that is offered within our programs. The training and quality assurance procedures described above could be implemented among groups of peer supporters within these organizations, with group supervision provided. mHealth approaches described above also could be leveraged to support the overall scalability of a peer support approach.

Finally, peer support is one way to address barriers to reaching medically underserved communities [65]. There is evidence of disparities in genetic testing access and uptake based on race, ethnicity, and socioeconomic status [66]. In addition, understanding the purpose and implications of genetic testing can be particularly challenging for patients with low health literacy [67, 68] and/or limited English proficiency [69, 70]. Concerted efforts are needed to decrease these disparities. If demonstrated to be effective in our ongoing studies, peer coaches could provide culturally appropriate information and help bridge the gap between medically underserved populations and genetic specialists. Training group members to support each other is consistent with principles of community-based participatory research [71], and has the potential for wide reach and long-term sustainability. An example of one such program is called “Árboles Familiares” (Family Trees) [72]. This program aims to train Latina community health workers and patient navigators to assess the risk of HBOC and provide appropriate referrals to at-risk Latina women. By engaging community members, programs such as these have the potential to address barriers to translating interventions into routine clinical and public health practice, and ultimately improve population health and reduce health disparities [73].

## Conclusions

Cascade testing approaches provide a tremendous opportunity to expand the impact of genetic counseling and testing. Outcomes would likely improve through continued efforts to apply this strategy using current methods. However, by applying novel delivery methods, such as peer support, we can likely amplify this effect. These efforts could be extended by clinicians partnering with local and national organizations supporting the hereditary cancer community to ensure ongoing training and education so that they can be even better prepared to serve those in need of support. This could include specific training in methods to support family communication and health decision making, leveraging key developmental windows.

In sum, the integration of peer supporters can allow for an extension of current clinical care models and supports provided by family and friends. While options for genetic testing are becoming more varied (e.g., direct to consumer, population-level) and complex (e.g., panels, exomes), greater demand for testing pushes the streamlining of counseling services (less frequent/intense, but equally effective). Offering peer supporters as adjuncts to care that do not overburden scarce genetic counseling resources is a novel delivery model for targeted support to those in need who can benefit most. Our novel methods enhance counseling’s longstanding focus on family communication and service utilization outcomes.

## Data Availability

Not applicable.
